# The Physician’s Dose and Symptom Book

**Published:** 1874-08

**Authors:** 


*The Physician's Dose and Symptom Book.* By Joseph H. Wythes, A. M., M. D. Philadelphia: Lindsay & Blakiston.

These books both seem to be of the labor saving variety, something after the style of the ready reckoners, etc. That they can be of much value to an educated physician we have much doubt, for they contain little if anything which he should not carry in his brain rather than in his pocket companion. As a ready means of gaining information upon some doubtful point they may be of value, but no physician should allow himself to rely upon such aids, and but few will take the trouble to carry them about in order to have them on hand in time of emergency.

